# The early advantage: How antenatal care shapes cognitive development in India: Evidence from Young Lives, India

**DOI:** 10.1371/journal.pgph.0004801

**Published:** 2025-11-26

**Authors:** Neha Adsul, Priya Rampal, Sarthak Gaurav

**Affiliations:** 1 Postdoctoral fellow at Centre for Policy Studies (CPS), Indian Institute of Technology (IITB), Bombay, India; 2 Senior Consultant-Quantitative Lead at Oxford Policy Management, Delhi, India; 3 Associate Professor, SJMSOM, Institute of Technology Bombay (IITB), India; McGill University, CANADA

## Abstract

It is universally accepted that early-life conditions significantly influence cognitive development in children; however longitudinal research from low- and middle-income countries remains limited. Antenatal care (ANC) as a critical early health-system contact, has the potential to reduce developmental disparities by promoting fetal brain development and improving pregnancy outcomes. Using data from the Young Lives study, this research examines developmental trajectories of 1,918 children, tracked from age 1–15 in India. Baseline maternal ANC data were collected when the child was one year old, while cognitive outcomes, measured through the Peabody Picture Vocabulary Test (PPVT) and Mathematics test scores, were assessed at ages 5, 8, 12, and 15. Multivariable regression models were used to examine associations between maternal ANC and cognitive outcomes, adjusting for key sociodemographic covariates. Propensity Score Matching minimized bias from observed confounders, and mediation analysis tested whether parental education or mid-day meal access explained observed pathways. The study found that children whose mothers accessed ANC regularly achieved significantly higher PPVT and Math scores at age 8, attenuating but persisting through ages 12–15. While mid-day meals showed no significant mediation, parental education consistently emerged as a strong positive predictor of children’s cognitive performance. Broader social determinants, such as caste, household wealth, sanitation, and access to clean water, were also significantly associated with disparities in test scores. These findings suggest that ANC functions not only as an indicator of maternal and child health but also as a potential contributor to sustained cognitive advantage during middle childhood. Strengthening ANC coverage and embedding it within multisectoral interventions in maternal health, education, nutrition, and social protection could enhance long-term developmental equity.

## 1 Introduction

A substantial body of research affirms that the foundations of a child’s physical and cognitive health, as well as their future success, are primarily established during early childhood [1,2]. Research into the associations between early life events and long-term outcomes reveals that the environment a child is born in plays a pivotal role in shaping the inputs and investments they receive during formative years. Failure to safeguard a child’s well-being during this critical developmental window can lead to lasting adverse effects on both physical and cognitive health. Emerging evidence further underscores that inequality takes root not only before a child enters school, but often begins even before birth [3,4]. These early-life inequalities are of particular concern for public health policy.

Despite global efforts to address the challenges of suboptimal early childhood development, it is estimated that over 200 million children under the age of five in low- and middle-income countries (LMICs) fail to achieve their cognitive, motor, and socio-emotional developmental potential due to inadequate nutrition, poor health, and poverty. In India, 35% of children under five are stunted, a statistic that points to chronic undernutrition. This concern is particularly acute given that undernutrition accounts for nearly half of all child deaths worldwide, underscoring the critical importance of early-life interventions that address both health and nutrition [5]. The conditions in India present a unique lens through which to explore how early interventions shape long-term trajectories.

Antenatal care (ANC) for pregnant mothers is one of the most effective strategies for mitigating such adverse childhood outcomes. ANC offers a vital opportunity to support fetal development, particularly during the critical period of in utero brain formation. Delivered by skilled health professionals, ANC encompasses nutritional guidance, medical interventions, fetal anomaly screenings, and counseling, each contributing to improved pregnancy outcomes and reducing the risk of low birth weight and preterm births [6].

Inadequate ANC, or its absence, has been linked to suboptimal cognitive development [7,8]. Cognitive skills, including measures such as Intelligence Quotient (IQ), are largely consolidated by ages 8–10, underscoring the critical importance of timely interventions to address developmental limitations during early childhood [9]. A meta-analysis of 48 studies across 20 developing countries has shown that maternal nutrition, including micronutrient supplementation during the first trimester, has significant benefits for early cognitive development [10]. Collectively, this body of literature underscores the importance of examining ANC as a critical early health-system touchpoint with potential implications for children’s subsequent cognitive development.

The longstanding debate over whether a child’s development is primarily shaped by genetic inheritance or environmental influences continues to evolve, with growing recognition of the complex interplay between biological influences and contextual factors. Genetic advances have demonstrated that environmental conditions can alter gene expression, challenging the long-held assumption that genetic endowment solely determines outcomes [11]. Studies have indicated that conducive environments can substantially reduce disparities in cognitive outcomes, such as IQ, among children of diverse racial or socioeconomic backgrounds. This is further supported by evidence suggesting that the quality of a child’s upbringing can, in many cases, exert a stronger influence on cognitive development than hereditary factors [12]. The emerging science of epigenetics underscores that disparities at birth are often not genetic but instead reflect complex interactions between genetic predispositions and environmental conditions [13]. Collectively, these findings underscore the policy salience of early-life interventions in LMICs, where addressing foundational disparities is critical to promoting equitable cognitive and health trajectories.

Environmental inputs, particularly those provided by parents, profoundly influence a child’s development. Heckman and Masterov [3] emphasize that targeted early life interventions can bring about positive changes in a child’s abilities. Deficiencies in cognitive skills resulting from early adverse conditions can be, partially or significantly, remedied by creating an enriched environment. Crucially, preventing developmental deficiencies during sensitive early childhood phases is far more effective than attempting to remediate them later. In developing countries, where baseline health indicators are lower and environmental shocks exert more severe and lasting effects, the stakes for early intervention are especially high [14]. In this context, ANC access may serve as a proxy for early engagement with health and social services.

Despite a growing body of research on the relationship between maternal ANC and child outcomes, significant knowledge gaps remain. Few studies in LMICs provide longitudinal evidence from the same cohort of children from early childhood into adolescence to assess whether ANC-associated advantages are sustained or attenuate over time [15]. Secondly, there has been relatively little work to integrate structural determinants (e.g., caste, poverty, sanitation, water) that may shape both access to ANC and cognitive outcomes in India. Finally, potential pathways, such as parental education and school-based nutrition, require closer analysis to understand how early advantages are associated with sustained developmental outcomes.

This study seeks to fill these gaps by examining the association between maternal ANC and children’s cognitive test scores, specifically the Peabody Picture Vocabulary Test (PPVT) and Math scores. Additionally, the analysis incorporates household, community, and maternal and child-level variables, drawing on the “fetal origins hypothesis”, which posits that early-life environmental exposures can shape adult health outcomes through complex gene-environment interactions [14]. By building on the findings of Cesare et al. (2013), who demonstrated significant relationships between ANC and children’s Cognitive Development Assessment (CDA) scores in several countries, including India, this study aims to provide insights into what constitutes an enabling environment for children’s growth [15]. By examining the relationship between cognitive achievement and environmental influences, this study offers critical insights for promoting skill development among children in developing countries. The findings underscore the importance of context-sensitive interventions that address structural inequities and support children’s developmental potential from the earliest stages of life [9].

This study contributes to existing research on the link between ANC and child development in three important ways: 1) longitudinal data spanning ages 5, 8, 12, and 15 were used to characterize age-patterned associations and their potential attenuation as the child progressed into adolescence; 2) the study complemented multilevel models with Propensity Score Matching (PSM) to reduce confounding from observed covariates, such as maternal education, household wealth, caste, gender, and place of residence. Analyses employed statistical techniques that account for baseline differences across groups; and 3) we examine plausible pathways via mediation (parental education; mid-day meals), and try to situate the results within India’s structural inequities. We posited a priori that access to ANC has a strong positive association with cognition in middle childhood (age 8), and diminished or null association by ages 12–15. We also posit that parental education would partially mediate later Math performance.

Overall, the study aims to examine the association between maternal ANC and children’s cognitive outcomes across developmental stages, situating ANC as an early-life health determinant within broader structural and educational contexts in under-resourced environments.

## 2 Conceptual framework

**Fig 1:**
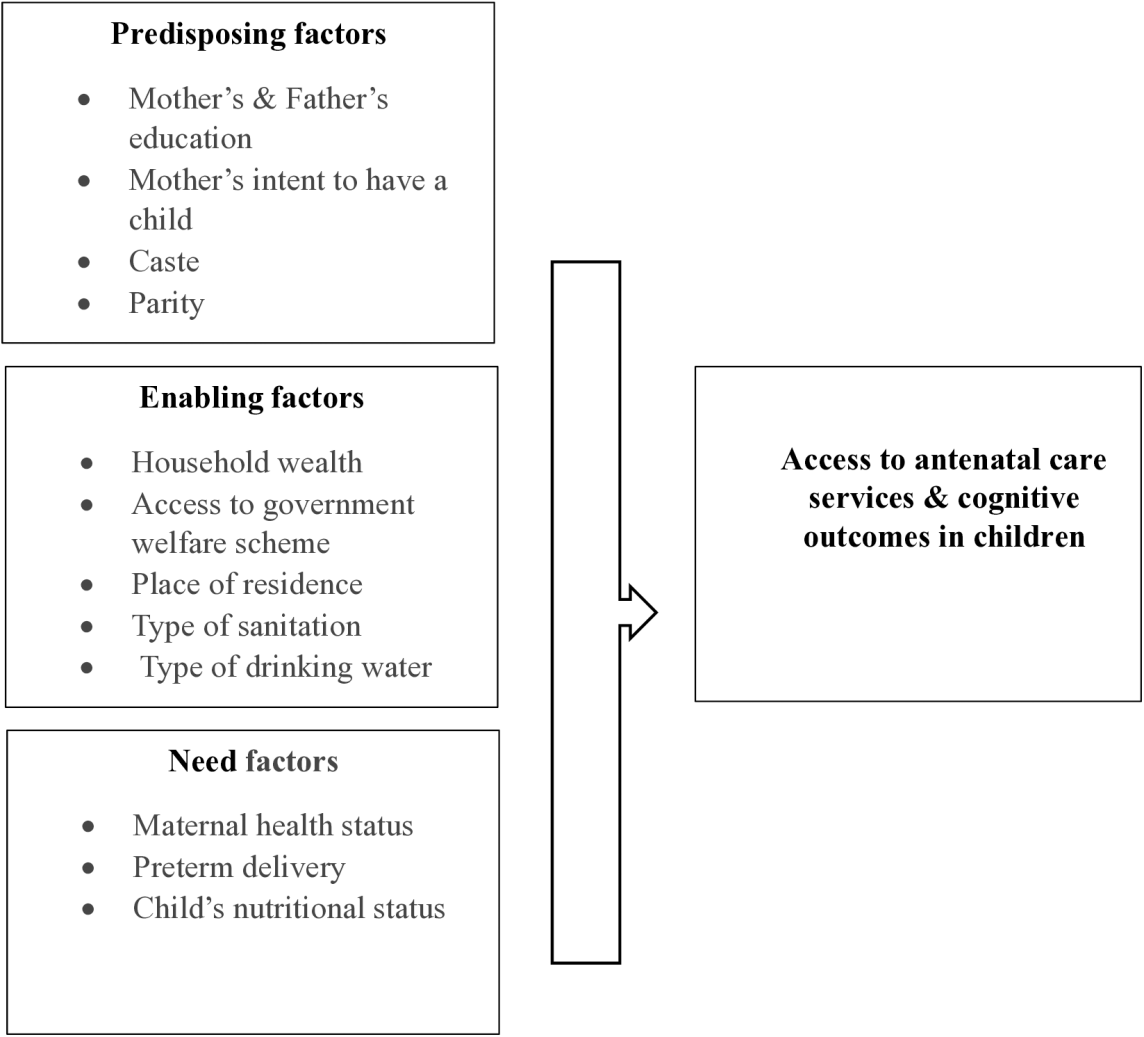
Modified conceptual framework for access to ANC and cognitive outcomes in children.

The conceptual framework for this study is shown in **Fig 1**.

Fig 1 illustrates a revised conceptual framework, adapted from Andersen’s Behavioral Model, that categorizes the primary determinants of ANC utilization into predisposing, enabling, and need-based domains. The predisposing domain encompasses maternal and paternal education levels, maternal intent regarding pregnancy, caste affiliation, and parity-related characteristics. The enabling domain includes household wealth, access to welfare schemes, sanitation, and water. The last domain, need-based, encompasses factors such as maternal health, preterm delivery, and child nutrition. Together, they affect ANC access, which in turn correlates with differences in cognitive development among children [16].

When applied to the context of maternal ANC and children’s cognitive development, the framework highlights the dynamic interrelationships among key determinants and their impact on child outcomes. The **predisposing factors**, which include individual and demographic characteristics, determine the likelihood of utilizing ANC services. Based on the analysis of secondary data and findings of prior research, maternal and paternal education, caste/social group, parity and maternal intent to have the child [17] were not found to have a direct association with accessibility to, or an immediate need for healthcare. Rather, they influence the disposition towards seeking care.

**Enabling factors** comprise the resources and contextual conditions that either facilitate or constrain access to healthcare services. They serve as pivotal mechanisms in translating predispositional characteristics into actual utilization of antenatal care. Key enabling factors in the study include the household wealth index, type of sanitation and drinking water facilities, place of residence [18], and access to welfare programs (e.g., mid-day meals) [19].

Enabling factors underscore the importance of structural and environmental support systems for ensuring equitable access to ANC. They determine whether women are predisposed to engage with (or discouraged from) utilizing ANC services, with downstream associations for early childhood development.

**Need-based factors** reflect both clinical health conditions and perceived requirements for care, exerting a more immediate influence on the demand for antenatal services. In this study, maternal health, i.e., maternal BMI (Body Mass Index), preterm delivery, child nutritional status (e.g., stunting), and perceived pregnancy risk’s function [20] as a direct catalyst for engaging with antenatal care.

### 2.1 Understanding the interplay of factors

The framework also emphasizes the dynamic interplay between these factors. Predisposing factors like education and caste influence the enabling environment by shaping socioeconomic conditions, which in turn determine the availability of resources, such as improved sanitation or welfare programs. These enabling conditions, in turn, intersect with perceived and actual health needs, forming a pathway through which maternal engagement with ANC is determined.

For instance, a low-income household, an enabling factor, may lack access to improved sanitation, thereby heightening maternal health risks (need-based factor). These compounded vulnerabilities can diminish the likelihood of seeking antenatal care, even when educational awareness (a predisposing factor) is present. This example underscores how structural constraints can override individual intent, reinforcing the importance of integrated interventions that address multiple determinants simultaneously.

The interconnectedness of the factors underscores the necessity of a multidimensional approach for improving ANC uptake. Such an approach would target structural, socioeconomic, and individual determinants. It further highlights the potential pathways for linking ANC utilization to a child’s cognitive outcomes, which are mediated by the broader social and environmental context.

Within this framework, we also consider plausible pathways that may link ANC to later cognition. Parental education has been consistently associated with cognitive outcomes in LMIC settings, both as a determinant of health-seeking behaviour and as a resource for children’s learning environments [21,22]. School-based nutrition schemes, such as India’s Mid-day Meal programme, have been shown to improve enrolment and indirectly support cognitive performance through better nutritional security [23,24]. Accordingly, parental education and school-based meal provision were identified a priori as the primary mediating pathways for empirical testing within this study.

## 3 Data and methods

### 3.1 Ethics statement

Ethical approval was not required for this study as only publicly available secondary data from the Young Lives dataset was used. The dataset was accessed from the website of the organization. No primary data was collected. As secondary user, we have strictly adhered to the terms and conditions set by Young Lives on their webpage, including ensuring participant confidentiality and anonymity (only anonymized data is available on the Young Lives website). The Young Lives dataset is openly accessible to all researchers for secondary research.

### 3.2 Data source and the study population

This study utilizes secondary data from the Indian sample of the younger cohort in the Young Lives longitudinal survey, encompassing children, their households, and communities. Data were collected across five survey rounds, conducted in 2002, 2006, 2009, 2013, and 2016, covering developmental trajectories and contextual influences. Data collection was undertaken in the states of Andhra Pradesh (Coastal Andhra and Rayalaseema regions) and Telangana. The ‘index child’, hereafter referred to by their age at each survey round, was aged, on average, 2, 5, 8, 12, and 15 years, during rounds 1–5, respectively.

Young Lives is a longitudinal study tracking the lives of approximately 12,000 children across four countries, India (Andhra Pradesh), Vietnam, Peru, and Ethiopia [25]. Initiated in 2002, the study follows two distinct cohorts in each country: the *Younger Cohort*, comprising around 2,000 children who were approximately two years old at baseline, and the *Older Cohort*, consisting of about 1,000 children aged around eight. Since its inception, the project has conducted five comprehensive rounds of data collection over 15 years, capturing rich, multi-level information on children, their households, and communities.

Since the aim of *Young Lives* study was to examine the causes and consequences of childhood poverty, and how policies may be associated with children’s well-being, the sample of the Young Lives project comprises primarily the poor. A detailed review of the questionnaires and datasets shows rich multidimensional information on the children’s lives. This includes household demographics, caregiver backgrounds and characteristics, and child-level attributes such as health, education, and psychosocial development. The sample reflects considerable diversity in terms of socioeconomic status, caste and social group affiliation, and living conditions, offering a depth of contextual data comparable to nationally representative surveys, while retaining the longitudinal advantage of tracking individual trajectories over time.

Although the sample of children is representative of the three regions of Andhra Pradesh, it is appropriate to analyze associations in similar contexts. One of the strengths of the YL study is that even by the fifth round, the overall attrition rate was only about 5 percent over nearly 15 years, which can be regarded as one of the lowest in longitudinal surveys of this nature [26].

For this paper, we restrict all analysis to the children from the younger cohorts, as a large set of information on child and maternal characteristics, vaccination status, and whether the mother received ANC is available only for this group. Other relevant covariates, including those related to early childhood cognitive development, which form the crux of our analysis, are available only for the younger cohort. These indicators reflect the initial developmental and contextual conditions of individual children and were used to frame the analysis of the Younger Cohort within the longitudinal study. Data were not available on child characteristics or important initial variables influencing the older cohort’s child growth and well-being.

### 3.3 Variables

#### 3.3.1 Dependent variable.

The dependent variable in this study comprises children’s cognitive test scores, specifically the Peabody Picture Vocabulary Test (PPVT) and Math assessments. The PPVT, first introduced in 1959, has been widely recognized as a robust measure of verbal ability and academic aptitude, and is often regarded as a benchmark indicator of general cognitive development [27]. The PPVT was adapted and standardized by YL researchers in each country. The younger cohort completed four PPVT tests in rounds two to five (at ages 5, 8, 12, and 15, respectively).

The objective of the test is to make a child identify one picture among a set of four presented that best corresponds to a word read out by the examiner. For example, in the PPVT, the recipient hears the word ‘bus’ in their mother tongue and is asked to identify one of four pictures corresponding to the word. The test is administered by arranging pictures in the ascending order of difficulty (17 sets of 12 items each). The starting set for the respondent child varies depending on her/his age. Test progress is computed by subtracting the errors from the scores earned by pointing out the exact item in the test, based on which the final PPVT score for an individual child was calculated [28].

Although concerns have been raised about the cultural validity of the Peabody Picture Vocabulary Test (PPVT), given its origins in Western contexts, the Young Lives research team undertook several adaptations to ensure its relevance and appropriateness for the study sample and local conditions. These efforts aimed to mitigate cultural bias and enhance the test’s applicability within diverse linguistic and socio-cultural settings.

Besides, pilot tests were conducted for validating the test instruments, and to ensure that they capture the relevant data they were intended to. The tests were first translated and then conducted in the children’s languages. Experts from diverse disciplines conducted item-by-item evaluations of the tests, applying multidimensional criteria such as gender sensitivity, social group neutrality, and cultural fairness. We used the raw PPVT scores for the analysis, which is in line with Cueto et al. [28], who argued that converting raw scores to standardized international PPVT scores is not necessary, as the objective here is to examine the differences in perceived ability among a cross-section of same-aged children. Since our approach does not involve benchmarking participants against an international standard, the raw PPVT score adequately serves the purpose of assessing verbal ability.

The Math skills were measured for the younger cohort of India when they were aged 8, 12, and 15 (rounds three to five). The test items were drawn from two international assessments: the Programme for International Student Assessment (PISA) and the Trends in International Mathematics and Science Study (TIMSS). The PISA items were used to evaluate applied problem-solving skills, while the ones from TIMSS measured curriculum-based numeracy. As age advanced, the test items became progressively more challenging at each point, aligning with expected developmental trajectories. Raw scores were converted into age-specific z-scores to allow comparison over time, and these standardized scores were used in our analyses [28,29].

PPVT and Mathematics scores were chosen as indicators of cognitive outcomes, as they are the only standardized assessments administered uniformly across all four rounds of the Young Lives survey. These tools are widely validated in LMIC contexts and serve as reliable proxies for verbal and numerical reasoning. While these outcomes may be influenced by formal schooling, our models adjust for enrolment status, maternal education, and household wealth to reduce confounding. Nonetheless, they should be interpreted only as proxies of verbal and numerical reasoning rather than as comprehensive measures of cognitive ability.

#### 3.3.2 Independent variable.

The key independent variable of interest in our paper is ‘mother’s access to ANC visits’, which has been examined as both a binary measure and a category. First, we used a binary variable from the Young Lives India dataset, which records whether the mother received any ANC during pregnancy (1 = Yes, 2 = No). This variable was used in two parts of our analysis: (i) in the PSM framework, where a binary treatment indicator is a methodological requirement, and (ii) in the multivariate regression models as a check for robustness as well as to explore whether the outcomes among children whose mothers utilized ANC differed from those with no ANC contact. The binary ANC variable captures an essential threshold in service engagement during the study period. This is of particular relevance in the Indian context, where a considerable proportion of women received no formal antenatal care. Furthermore, incorporating this binary indicator in regression models allows us to understand whether child outcomes were statistically associated with basic ANC access.

The study also used a composite ordinal variable, which was constructed by the Young Lives research team [30]. A three-point scoring algorithm was used: (i) timing of the first ANC visit (before the fourth month), (ii) total number of visits (five or more), and (iii) receipt of tetanus toxoid (TT) injections. The child’s mother received one point for each of these criteria, yielding a score between 0 and 3. A score of 0 indicates no utilization of ANC, while scores of 1–3 reflect increasing levels of ANC service engagement. The scores were coded into four ordered categories: No ANC (0), Low Utilization (1), Medium Utilization (2), and High Utilization of ANC (3). In our regression models, this variable allowed us to link graded associations with cognitive outcomes.

The use of both binary and categorical ANC variables serves complementary purposes: the binary indicator identifies the presence or absence of ANC contact (critical for public health decision-making), while the categorical indicator enables a nuanced understanding of whether increased intensity of ANC utilization is statistically associated with differences in children’s cognitive outcomes. It is important to acknowledge that this variable reflects only the quantity and timing of service exposure; it does not encompass qualitative aspects such as provider type, counseling, or the nature of clinical care delivered.

#### 3.3.3 Other covariates.

We have included other relevant independent variables. These are based predominantly on the theoretical aspects of, and empirical research, on maternal and child health. Maternal education, father’s education, [15], the sex of the child, place of residence, type of birth (pre- or full-term), maternal Body Mass Index (BMI), maternal willingness to have the child [31,32], caste [33,34], school enrolment status of the child, number of siblings alive, access to mid-day meals (feeding programs in school), and anthropometric status [23].

In addition to the key independent variable, the following independent variables were incorporated into the analysis: the level of ANC services initiated in the first trimester, based on the WHO’s 2016 recommendations [6]. This variable was categorized into four levels of antenatal care:

- **None**: No ANC received.
- **Low**: A single ANC visit conducted at or before four months of pregnancy.
- **Medium**: Multiple ANC visits recorded between the fifth and ninth months of pregnancy.
- **High**: At least five ANC visits spread across the nine months, along with completion of tetanus toxoid (TT) immunization.

Other variables included household wealth (as provided in the Young Lives dataset), which is a composite wealth index of housing quality, household durables, and facilities. The index can take a value between 0 and 1. For this study, we used the index by categorizing it into quintiles to facilitate empirical analysis, treating it as one of the regressors in our model.

Additionally, we included variables that capture the type of sanitation and drinking water facilities. We classified toilet facilities based on two main categories, ‘improved’ and ‘unimproved’, as defined by the World Health Organization (WHO) and UNICEF [35]. These categories are based on the toilets’ ability to separate human waste from human contact hygienically. ‘Improved sanitation facilities’ include flush toilets, piped sewer systems, septic tanks, and ventilated improved pit latrines. ‘Unimproved sanitation facilities’ include pit latrines without a slab, bucket toilets, and open defecation, as they do not adequately separate waste from human contact. Similarly, based on their safety and reliability, we classified the drinking water sources into ‘improved’ and ‘unimproved’ categories. ‘Improved drinking water sources’ include piped water, boreholes, and protected wells, as they are likely to be safe from contamination. ‘Unimproved drinking water sources’ include unprotected wells, surface water, and tanker trucks, all of which are more prone to contamination and hence are not considered safe for consumption.

### 3.4 Approach to analysis

The analytical approach for this study followed three key steps:

a. Descriptive statistics: Frequency distributions and summary statistics were computed to examine the study population’s background characteristics. By focusing on maternal, child, and household variables, the descriptive analyses provided an understanding of the conditions influencing cognitive development.
b. Multilevel regression model: Multilevel regression was employed to explore the association between ANC access and children’s cognitive outcomes. By accounting for the nested nature of the data, this method allowed for a nuanced analysis that integrates variables from individual, household, and community contexts. Separate models were estimated for cognitive scores, Peabody Picture Vocabulary Test (PPVT) and Math scores, at different ages (5, 8, 12, and 15 years).
c. Propensity Score Matching: PSM was employed using nearest neighbor matching with three neighbors to estimate the Average Treatment Effect on the Treated (ATET). The propensity score model incorporated key covariates, such as the child’s sex, maternal intent to conceive, preterm birth status, and caste group. Covariate balance was assessed by directly computing Standardized Mean Differences (SMDs) in STATA. In addition, mediation analysis was done (see Section 4.4) to explore the potential pathways linking ANC with cognitive outcomes.

The Variance Inflation Factor (VIF) assessed multicollinearity among independent variables. All variables exhibited a VIF below the threshold of 2, indicating no significant multicollinearity issues. Data analysis was performed using STATA Version 17.

The study adheres to the Strengthening the Reporting of Observational Studies in Epidemiology (STROBE) guidelines for cohort studies. A completed STROBE checklist detailing where each item is addressed in the manuscript is provided as **S1 Table**.

## 4 Results

### 4.1 Demographic characteristics

**Table 1** presents a statistical summary and brief overview of the key variables used in the study, which focus on maternal, child, and household characteristics.

**Table 1:** Summary statistics for variables of interest.

Variable	Mean	Std. deviation	Percentage	No. of observations
**Access to ANC**				
Have access			88.11	1985
No access			11.89	
**Level of antenatal care**				
None			12.67	
Low			22.13	1957
Medium			22.64	
High			42.57	
**Sex**				
Female			46.25	2011
Male			53.75	
**Place of residence**				
Urban			25.26	2011
Rural			74.74	
**Birth type**				
Full-term			90.59	1923
Pre-term			9.41	
**Maternal BMI**	18.24	6.95		
**Mother’s Intent to have the Child**				
Intended birth			91.93	1970
Unintended birth			8.07	
**Mother’s Education**				
Primary or Higher Levels			39.67	2009
Incomplete Education			60.33	
**Father’s Education**				
Primary or Higher Levels			19.21	1947
Incomplete Education			80.73	
**Child’s Enrolment in Pre-school**				
Enrolled			57.05	1695
Not enrolled			42.95	
**Enrolment in School**				
*At Age 8*				
Enrolled			99.01	1921
Not Enrolled			0.99	
*At Age 12*				
Enrolled			97.18	1912
Not Enrolled			2.82	
*At Age 15*				
Enrolled			91.13	1838
Not Enrolled			8.87	
**Number of siblings**				
Young Child is the Only Child			37.64	2011
Up to Three Children			1055	
More than Three Children			199	
**Mid-day meal**				
*At Age 5*				
Available			34.16	1947
Unavailable			65.84	
*At Age 8*				
Available			50.73	1922
Unavailable			49.27	
*At Age 12*				
Available			98.69	1910
Unavailable			1.31	
*At Age 15*				
Available			98.42	1893
Unavailable			1.58	
**Anthropometry**				
Stunted			31.03	2011
Normal			68.97	
**Caste (social group) of Child**				
Schedule caste			18.40	2011
Schedule tribe			14.57	
Backward class			45.95	
Open/Other castes			21.08	
**Household Wealth Index**	2.99	1.41		2006
**Toilet Quality**				
Improved			70.07	2008
Unimproved			29.93	
**Drinking Water Quality**				
Improved			16.21	2011
Unimproved			83.79	
PPVT Scores At Age 5	27.44	21.12		1851
PPVT Scores At Age 8	58.48	30.45		1901
PPVT Scores At Age 12	43.06	7.83		1903
PPVT Score At Age 15	47.35	7.88		1890
Math Score at Age 8	12.02	6.42		1904
Math Score at Age 12	12.76	6.60		1858
Math Score at Age 15	10.30	5.12		1840

Table 1 presents descriptive statistics that provide a foundational overview of key demographic, socioeconomic, and developmental attributes of the sample for interpreting subsequent analyses. Most mothers (88.11%) had access to Antenatal care, while 11.89% reported no access. Among the mothers who accessed ANC, 42.57% utilized high-level services, whereas 22.64% and 22.13% accessed medium- and low-level care, respectively. Maternal education levels highlight a notable disparity: while 39.67% of mothers had completed primary or higher education, a majority, 60.33%, had not. This educational gap is even more pronounced among fathers, with only 19.21% reaching primary or higher levels, underscoring a broader trend of limited formal schooling among the parents. Preschool enrollment stood at 57.05%, increasing to near-universal levels at age 8 (99.01%) before declining modestly to 91.13% by age 15.

In the sample, 46.25% were female and 53.75% male. Urban residence comprised 25.26% of the sample, whereas rural settings dominated at 74.74%. Full-term births were predominant (90.59%) in the sample, and mean maternal BMI was 18.24 (SD: 6.95).

Household indicators reveal substantial variability, with the mean wealth index at 2.99 (SD: 1.41). While most households (70.07%) reported access to improved sanitation, only 16.21% had access to improved drinking water. Anthropometric data indicated that 31.03% of children were stunted.

Cognitive outcomes, assessed through PPVT and Math scores, showed developmental trends. The mean PPVT scores were 27.44 (SD: 21.12) at age 5, which peaked at 58.48 (SD: 30.45) by age 8 and then stabilized at 47 by age 15. Math scores followed a similar pattern: 12.02 (SD: 6.42) at age 8 and then declining to 10.30 (SD: 5.12) by age 15.

Caste-wise distribution reveals that 45.95% of children belonged to backward classes, indicating a significant representation from historically marginalized groups. Scheduled Castes and Scheduled Tribes comprised 18.40% and 14.57% of the sample, respectively, further underscoring the socio-cultural diversity and structural inequalities embedded within the cohort. Access to midday meals showed a substantial increase from 34.16% at age 5 to over 98% at ages 12 and 15 years.

**Fig 2:**
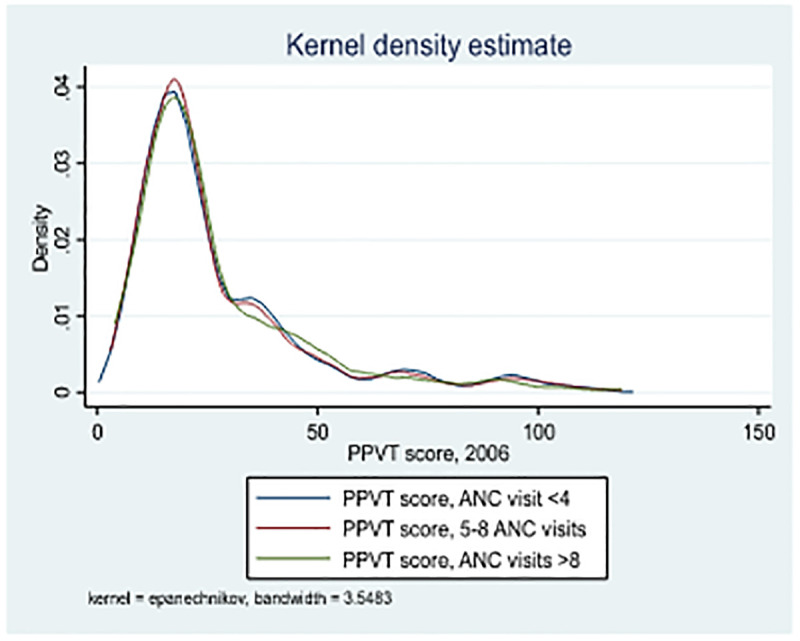
Kernel regression of ANC visits and PPVT scores among children.

Fig 2 shows a non-parametric kernel regression examining the relationship between children’s cognitive scores, measured via the Peabody Picture Vocabulary Test (PPVT), on the x-axis, and the number of maternal ANC visits on the y-axis. By avoiding assumptions of linearity, the plot provides a nuanced visualization of how maternal engagement with prenatal care may be linked to early cognitive development. Data were drawn from the Young Lives longitudinal survey in India; ANC was recorded in Round 1, and PPVT scores were collected at ages 5, 8, 12, and 15. After eight visits, the cognitive benefits tend to plateau, suggesting diminishing returns in terms of observable gains in the scores.

**Fig 3:**
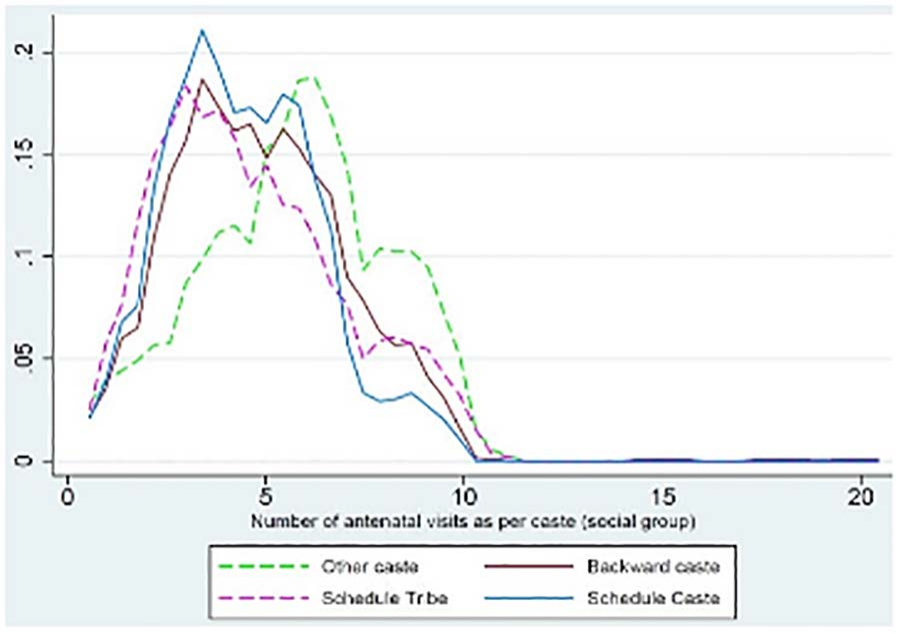
Kernel regression of maternal caste group and antenatal care visits.

Fig 3 presents a non-parametric kernel regression examining the relationship between maternal caste or social group (y-axis) and the number of ANC visits received (x-axis). Visualizing social gradients in ANC utilization helps illustrate how structural factors, such as caste stratification, influence access to maternal health services. The use of kernel regression enables the detection of non-linear trends in the data.

These patterns reveal significant disparities in ANC utilization by caste group. It is seen that while most women from the open have accessed more than five ANC visits, access for those from marginalized groups, the Scheduled Tribes (STs), Scheduled Castes (SCs), and Other Backward Castes (OBCs), tends to cluster around zero to five visits, highlighting persistent inequities in maternal healthcare access.

### 4.2 Association of maternal access to ANC with children’s cognitive test scores

The regression analysis in Table 2 examines the association between the independent variables, including maternal access to ANC services, on children’s cognitive outcomes at different ages (5, 8, 12, and 15 years), as indicated by the PPVT and Math test scores. The figures in the table indicate a significant positive association between maternal access to ANC and children’s cognitive scores, particularly in middle childhood (age 8).

**Table 2:** Multilevel regression model for the association between mother’s antenatal care access and children’s cognitive outcomes.

	Age 5	Age 8	Age 12	Age 15	Age 8	Age 12	Age 15
	PPVT_score	PPVT_score	PPVT_score	PPVT_score	Math_score	Math_score	Math_score
**Access to ANC services**							
**(No access)**							
**Accessed**	**1.17**	10.26***	**0.93**	**0.66**	1.22***	**0.31**	**0.15**
	**(1.69)**	**(2.18)**	**(0.58)**	**(0.6)**	**(0.46)**	**(0.5)**	**(0.39)**
**Access to ANC services** **(No access)**							
**Low**	**.92**	9.47***	**.75**	**.92**	**.75**	**.58**	**.16**
	**(1.93)**	**(2.52)**	**(.66)**	**(.67)**	**(.52)**	**(.56)**	**(.44)**
**Medium**	**1.03**	9.4***	**.84**	**-.53**	1.12**	**.31**	**.02**
	**(1.95)**	**(2.56)**	**(.67)**	**(.68)**	**(.53)**	**(.57)**	**(.45)**
**High**	**1.2**	11.13***	1.37**	**.44**	1.7***	**.2**	**-.08**
	**(1.84)**	**(2.39)**	**(.63)**	**(.64)**	**(.49)**	**(.54)**	**(.42)**
**Sex**							
**Male**							
**Female**	**.43**	-7.07***	.63*	**.11**	**-.37**	**-.13**	**-.02**
	**(1.09)**	**(1.46)**	**(.37)**	**(.38)**	**(.3)**	**(.32)**	**(.25)**
**Place of residence**							
**Urban**							
**Rural**	**1.81**	-4.76**	-1.62***	**-.09**	**.65**	-.82*	**.19**
	**(1.82)**	**(2.06)**	**(.59)**	**(.54)**	**(.46)**	**(.48)**	**(.35)**
**Birth type**							
**Full-term**							
**Pre-term**	**-.36**	-7.15***	**-.84**	**-.47**	**-.39**	**-.37**	**-.71**
	**(1.88)**	**(2.47)**	**(.64)**	**(.67)**	**(.51)**	**(.56)**	**(.43)**
**Maternal BMI**							
**Normal range**							
**Below or above normal**	**-.71**	-7.46***	-1.46***	**.57**	-.99***	.75**	.75***
	**(1.22)**	**(1.56)**	**(.41)**	**(.41)**	**(.32)**	**(.35)**	**(.27)**
**Maternal intent to have the child**							
**Intended birth**							
**Unintended birth**	**2.91**	**-3.45**	**-.38**	**-1.07**	**-.83**	**-.51**	**.16**
	**(2.02)**	**(2.69)**	**(.7)**	**(.71)**	**(.55)**	**(.6)**	**(.46)**
**Mother’s education**							
**Primary or higher levels complete**							
**Incomplete schooling**	**.35**	-7.62***	-.86*	**.16**	-2.81***	-1.21***	**.18**
	**(1.38)**	**(1.82)**	**(.48)**	**(.48)**	**(.38)**	**(.41)**	**(.31)**
**Father’s education**							
**Primary or higher levels complete**							
**Incomplete schooling**	**.54**	-2.95*	**-.43**	**.34**	-.98***	**0**	**-.05**
	**(1.28)**	**(1.71)**	**(.44)**	**(.44)**	**(.35)**	**(.37)**	**(.29)**
**School enrolment**							
**Enrolled**	-6.89***	**-10.09**	**-.31**	-4.02***	-5.72***	**-.29**	-2.3***
**Not enrolled**	**(1.68)**	**(8.11)**	**(1.14)**	**(.68)**	**(2)**	**(.97)**	**(.45)**
							
**Number of siblings alive**							
**Only child**							
**Up to three children**	**.2**	-4.81***	**-.51**	**.09**	-1.04***	**-.4**	**.11**
	**(1.34)**	**(1.76)**	**(.46)**	**(.46)**	**(.36)**	**(.39)**	**(.3)**
**More than three children**	**5.2**	**-8.95**	**-.35**	**-.05**	**-1.16**	**-.85**	-1.91*
	**(4.7)**	**(5.82)**	**(1.52)**	**(1.59)**	**(1.19)**	**(1.33)**	**(1.05)**
**Access to Mid-day meal**							
**No access**							
**Had access**	7.86***	**1.65**	**.52**	**1.12**	**.42**	**1.96**	**.29**
	**(1.77)**	**(1.72)**	**(1.53)**	**(1.56)**	**(.36)**	**(1.35)**	**(1.03)**
**Anthropometry**							
**Normal**							
**Stunted**	-6.98***	-6.12***	-2.68***	-1.02**	-1.75***	-1.83***	-1.23***
	**(1.14)**	**(1.63)**	**(.41)**	**(.42)**	**(.33)**	**(.35)**	**(.28)**
**Caste of child**							
**Open caste**							
**Backward caste**	**-.74**	-5.03**	**-.4**	**-.07**	-.71*	**-.29**	**.04**
	**(1.52)**	**(2.02)**	**(.52)**	**(.53)**	**(.41)**	**(.45)**	**(.35)**
**Schedule caste**	**-.8**	**-2.94**	**.01**	**-.28**	-1.12**	**-.26**	**-.67**
	**(1.86)**	**(2.46)**	**(.64)**	**(.64)**	**(.5)**	**(.55)**	**(.42)**
**Schedule tribe**	-5.35**	-6.4**	**-.49**	**-1.06**	-3.18***	-1.16*	**-.27**
	**(2.08)**	**(2.72)**	**(.72)**	**(.73)**	**(.56)**	**(.62)**	**(.48)**
**Household wealth category**							
**Rich**							
**Average**	-3.67**	**.29**	**.33**	**.43**	**.45**	**.12**	**-.41**
	**(1.5)**	**(2)**	**(.51)**	**(.54)**	**(.41)**	**(.43)**	**(.35)**
**Poor**	-3.29**	**-1.68**	**-.57**	-1.39**	**-.35**	**-.37**	**-.48**
	**(1.64)**	**(1.85)**	**(.56)**	**(.54)**	**(.34)**	**(.46)**	**(.35)**
**Type of sanitation facility**							
**Improved**							
**Unimproved**	**-.87**	**.47**	**-.81**	**-.84**	**-.1**	-2.18***	-.71**
	**(1.97)**	**(2.05)**	**(.58)**	**(.53)**	**(.44)**	**(.35)**	**(.34)**
**Type of drinking water facility**							
**Improved**							
**Unimproved**	-3.69**	**-3.6**	-3.73**	**-1.86**	-1.12*	**1.14**	-2.57*
	**(1.62)**	**(3.27)**	**(1.82)**	**(2.04)**	**(.67)**	**(1.54)**	**(1.32)**
							
**_cons**	26.62***	77.15***	42.87***	51.34***	16.71***	15.14***	13.67***
	**(3.23)**	**(4.89)**	**(2.04)**	**(2.2)**	**(.94)**	**(1.7)**	**(1.42)**
**Observations**	**1454**	**1645**	**1660**	**1672**	**1650**	**1620**	**1639**
**R-squared**	**.07**	**.13**	**.07**	**.04**	**.17**	**.08**	**.05**

***Standard errors are in parentheses***

*** p < .01, ** p < .05, * p < .1

These findings are of relevance considering that:

(a) While 57% of the children were enrolled in preschool by age 5, by age 8 the enrolment was 99%.
(b) Many cognitive assessments mirror traditional school-based testing formats, such as the paper-and-pencil methods commonly used in Indian classrooms. These formats align with cultural practices familiar to children who have spent time in formal schooling. In contrast, children with limited exposure to such environments often underperform, not due to a lack of ability, but because the testing context is unfamiliar. A similar pattern was documented among Brazilian street children, who demonstrated remarkable competence in real-world problem-solving but struggled with standardized test formats [36,37].

Thus, while these tests may be reliable among school-going children, they may not fully capture children’s abilities with less exposure to formal schooling.

#### 4.2.1 Maternal access to ANC services and cognitive scores in children.

As noted earlier, the multilevel regression results in Table 2 reveal a significant link between maternal access to ANC services and children’s cognitive outcomes, with the strongest effects observed during middle childhood (age 8). At this developmental stage, the positive association is most pronounced for both PPVT (Peabody Picture Vocabulary Test) and mathematics scores, indicating that maternal access to ANC may be significantly correlated with enhanced cognitive performance. Children whose mothers had access to ANC services exhibited significantly higher cognitive scores at age 8 compared to children whose mothers had no access to ANC. For example, the PPVT score at age 8 was 10.26 points higher (p < 0.01) for children whose mothers had access to ANC compared to those who did not. The corresponding difference in Math score was 1.22 points (p < 0.01) (Table 2).

The analysis of ANC levels (low, medium, and high access) reinforces these findings. Children whose mothers had ‘medium-level ANC access’ (5–8 visits) showed significant gains in cognitive outcomes, particularly at age 8, where their PPVT and Math scores were, respectively, 9.4 points and 1.12 points (p < 0.05) higher compared to their peers who had no ANC access.

The most pronounced cognitive gains were observed among children whose mothers had high-level access to Antenatal care, which included receiving tetanus toxoid (TT) injections. At age 8, this group had 11.13 points higher PPVT scores (p < 0.01) and 1.7 points higher Math scores (p < 0.01) than their peers, indicating that comprehensive antenatal care, including TT injections, was statistically associated with higher cognitive scores.

#### 4.2.2 Gender disparities in cognitive outcomes.

The regression results also highlight significant gender disparities in cognitive scores. At age 8, female children had lower PPVT scores than males (β = -7.07, p < 0.01), a trend that persisted at age 12. Though the effect on Math scores for females was insignificant, it remained negative across different age groups.

#### 4.2.3 Place of residence.

The place of residence was another significant predictor of cognitive outcomes, particularly in vocabulary skills. Children in rural areas had lower PPVT scores at age 8 (β = -4.76, p < 0.05) and age 12 (β = -1.62, p < 0.01), while their Math scores were less affected.

#### 4.2.4 Birth type and maternal BMI.

Preterm birth and maternal abnormal BMI, defined as either underweight or overweight, were both significantly associated with lower cognitive scores. At age 8, preterm children scored 7.15 points lower on the PPVT (p < 0.01), while children of mothers with abnormal BMI showed a negative association with cognitive outcomes (β = –7.46, p < 0.01).

#### 4.2.5 Maternal intent to have a child.

The maternal intent to have the child has a less overt, though still important, association with cognitive outcomes. While the effects of the PPVT scores are not statistically significant, extant literature suggests that unwanted pregnancies are often associated with lower maternal engagement, which can translate into poorer health and cognitive outcomes for the child. Maternal disengagement stemming from unintended pregnancies usually correlates with compromised nutrition and emotional neglect, especially in the early years of life, when such factors are most critical for cognitive development [38].

#### 4.2.6 Maternal and paternal education.

Both maternal education and household wealth were found to be positively associated with children’s cognitive scores. As Table 2 shows, children whose parents did not complete primary school exhibited significantly lower cognitive abilities between ages 8 and 12.

#### 4.2.7 School enrollment and access to mid-day meals.

The cognitive disadvantage of being out of school persists across ages, with lower performance on both PPVT and Math tests, indicating the critical association between school attendance and cognitive skills. Children who were not enrolled in school had significantly lower cognitive scores across multiple ages, particularly at ages 5, 8, and 15 for both PPVT and Math scores. At age 5, children not enrolled in school scored significantly lower on cognitive assessments. Their PPVT scores were 6.89 points lower (p < 0.01) than those of enrolled peers, and a similar trend was observed in Math scores, with a 4.02-point deficit (p < 0.01). A similar trend was observed among children at age 15. Moreover, children with no access to mid-day meals had significantly lower PPVT and Math scores at age 5 (β = -7.86, p < 0.01) and age 8, highlighting the association between school-based nutrition programs and cognitive outcomes. Not having access to mid-day meals consistently predicts lower cognitive scores in children until age 15.

#### 4.2.8 Number of siblings.

The analysis shows that children with more siblings, particularly those having more than three, tend to have lower cognitive outcomes compared to those who are the only child of their parents. For instance, at age 8, children with more than three siblings had a PPVT score that was 8.95 points lower. However, this result is not statistically significant. Similarly, Math scores were affected negatively at age 15, a drop of 1.91 points (p < 0.1).

#### 4.2.9 Stunting and cognitive development.

Childhood stunting consistently predicted negative cognitive outcomes from age 5–15. Stunted children had significantly lower PPVT and Math scores throughout, indicating the strong association between nutrition and cognitive development.

#### 4.2.10 Caste/Social group and cognitive outcomes.

Caste emerged as a significant predictor of cognitive disparities, with children from Scheduled Tribes (STs) and Scheduled Castes (SCs) scoring markedly lower on cognitive assessments at ages 8 and 12, relative to children from higher caste groups.

#### 4.2.11 Household wealth status.

Household wealth status was positively associated with cognitive outcomes. The results indicate that wealthier children performed better on both PPVT and Math tests. Children from poor households scored 3.29 points lower (p < 0.05) on the PPVT at age 5 than those from wealthier households. Household wealth status was a consistently negative predictor of cognitive scores of children from average and poor households until they were 15.

#### 4.2.12 Type of sanitation and drinking water facilities.

Children living in households with unimproved sanitation consistently exhibited lower cognitive performance. At age 12, their Math scores were 2.18 points lower (p < 0.01) compared to peers with improved sanitation, and this disadvantage persisted through age 15.

Similarly, children from households with unimproved drinking water sources showed lower cognitive performance. At age 5, their PPVT scores were 3.69 points lower (p < 0.05) compared to those with access to better water facilities, with this gap persisting into later ages. At age 15, their Math scores were 2.57 points lower (p < 0.1). Poor water quality increases the risks of waterborne diseases, affecting children’s physical health, school attendance, and cognitive development [39–41].

### 4.3 Long-term cognitive effects of antenatal care: A propensity score analysis

For a better understanding of the association between maternal access to ANC and children’s cognitive outcomes, we employed PSM to reduce selection bias based on observable characteristics. PSM facilitates a balanced comparison between children whose mothers had access to ANC and those without, by adjusting for covariates that may influence both ANC uptake and child development. Using nearest neighbor matching with three neighbors, we estimated the Average Treatment Effect on the Treated (ATET), as discussed in the Methods section.

Covariate balance was assessed using Standardized Mean Differences (SMDs) calculated in STATA, all of which were below the conventional threshold of 0.1, indicating satisfactory post-match balance. While this approach reduces bias from observed confounders, we acknowledge that unobserved heterogeneity and the observational nature of the data limits the ability to draw causal inferences. The results of the matched analysis are presented below for PPVT and Math scores at ages 5, 8, 12, and 15.

Table 3 reports the Average Treatment Effect on the Treated (ATET) using PSM. The estimates reflect the average difference in cognitive scores associated with maternal ANC access for children whose mothers received ANC (treated) compared to a matched control group. By balancing observed characteristics between treated and untreated groups, PSM reduces confounding and strengthens the internal validity of our findings [42].

**Table 3:** Average treatment effect on treated (ATET) estimates for children’s cognitive outcomes by age.

	Average treatment effect on treated			
Children’s cognitive outcomes	Coef.	SE	t-value	p-value
PPVT score at age 5	0.28	1.58	0.18	0.8
PPVT score at age 8	9.53^***^	1.89	5.04	0.0
PPVT score at age 12	0.08	0.57	0.13	0.8
PPVT score at age 15	-0.35	0.61	-0.57	0.5
Math score at age 8	1.35^***^	0.5	2.70	0.0
Math score at age 12	0.33	0.53	0.62	0.5
Math score at age 15	0.23	0.41	0.55	0.5

Significance: *** p < .01, ** p < .05, * p < .1.

To examine the differential effects of maternal access to ANC on children’s cognitive outcomes, propensity scores were estimated using logit models based on key explanatory covariates. These included maternal education, caste, household wealth, the child’s gender, and place of residence, factors empirically and theoretically linked to both access to ANC and child development. For example, maternal education is associated with health-seeking behaviors [21,43], while household wealth is associated with access to care and early childhood resources [44]. Caste, a key structural determinant in the Indian context, determines access to services due to entrenched social stratification [33,34]. Gender norms, such as son preference, affect maternal investments in health [45,46], and the place of residence (urban vs. rural) influences service availability [47,48]. Consistent with existing literature, the selection of covariates enhances the internal validity of our matched estimates by ensuring comparability between treatment and control groups.

The analysis reveals that children whose mothers received ANC had statistically higher scores at specific ages. At age 8, on average, these children scored 9.5 times higher on the PPVT and 1.29 points higher in Math (p < 0.01) than those in the matched comparison group. This pattern is consistent with our multilevel regression results. This supports the finding that maternal access to ANC is positively associated with better cognitive scores in the early years of schooling.

In contrast, no statistically significant differences were observed at later ages. At ages 12 and 15, no statistically significant associations were found between ANC access and cognitive scores. For example, the coefficients for PPVT and Math scores at ages 12 and 15 are not statistically significant (e.g., PPVT at age 12: coef. = 0.08, p = 0.8; Math at age 12: coef. = 0.33, p = 0.5). By age 15, the differences are negligible (PPVT coef. = –0.35, p = 0.5; Math coef. = 0.23, p = 0.5). It is important to stress that, while PSM enhances group comparability and mitigates selection bias from observable covariates, it does not address unobserved confounding. Consequently, the findings should be interpreted as statistical associations rather than definitive causal effects.

### 4.4 ANC and environmental factors working in synergy

While our results show a significant association between maternal access to ANC and improved cognitive outcomes in early childhood, particularly for mathematical reasoning at age 8, these associations appear to weaken by adolescence, suggesting a diminishing effect over time to explore this phenomenon more deeply, we conducted mediation analyses at age 15, when cognitive scores exhibit high stability [49], to examine whether specific factors help explain the pathways linking ANC to later cognitive outcomes.

Mediation analyses were restricted to parental education and mid-day meal access, covariates identified a priori from the literature [21,29] as plausible pathways. Other covariates reflecting environmental and socioeconomic characteristics were treated as structural background factors and included in regression models as confounders, rather than as mediators.

Our mediation analysis indicates that the positive association between maternal ANC access and Math scores at age 15 is significantly mediated by both maternal and paternal education levels. The Natural Indirect Effect (NIE) through maternal education was 0.103 (95% CI: 0.002, 0.205), with a corresponding Natural Direct Effect (NDE) of 1.144 (95% CI: 0.315, 1.971) and Total Effect (TE) of 1.246 (95% CI: 0.397, 2.096). Similarly, paternal education showed an NIE of 0.120 (95% CI: 0.012, 0.228), an NDE of 1.246 (95% CI: 0.399, 2.093), and a TE of 1.366 (95% CI: 0.515, 2.217).

In contrast, no significant mediation effects were observed for PPVT scores at age 15, nor through access to mid-day meals. For example, the NIE via maternal education on PPVT was 0.005 (95% CI: −0.078, 0.088), and via paternal education was −0.028 (95% CI: −0.193, 0.137), suggesting no meaningful indirect influence through these channels (**S2 Table**).

## 5 Discussion and policy directions

To our knowledge, no previous published analyses from the *Young Lives India* cohort have examined the association between maternal ANC and children’s cognitive outcomes across four developmental stages, spanning early childhood to adolescence. This study found that maternal access to ANC was positively associated with children’s cognitive outcomes, particularly in middle childhood. The most consistent differences appeared at age 8, when children whose mothers accessed ANC had higher PPVT and Mathematics scores. Analysis also showed that the strength of these associations diminished by ages 12 and 15. These findings are consistent with research emphasizing the role of maternal health and preventive care in supporting early child development [43,50]. The attenuation observed in adolescence suggests that the influence of ANC is most visible during early schooling, a period marked by the consolidation of foundational cognitive skills [36,37].

Gender disparities were particularly evident in vocabulary scores, with girls underperforming relative to boys. This mirrors broader structural inequalities in South Asia, including unequal household labor burdens, son preference, and gendered patterns of food allocation [46,51–54]. Rural children performed worse in vocabulary skills, likely due to disparities in educational quality and access to stimulating environments [47,48]. Pre-term birth and maternal abnormal BMI were also linked with lower scores, underscoring the importance of maternal health and nutrition during pregnancy [43,55,56]. Unintended pregnancies showed association with poorer cognitive outcomes, though such estimates may reflect reporting and rationalisation biases [38,57,58].

Parental education was a consistent predictor of cognitive performance, which aligns with evidence that educated caregivers support children’s nutrition, health, and learning environments. School enrolment and access to mid-day meals were positively associated with cognitive scores, consistent with literature linking formal education and school feeding to improved learning outcomes [21,23,24]. Larger sibships were linked with lower performance, a likely indication of resource dilution effects [59,60]. Stunted children consistently underperformed, reaffirming the strong link between physical growth and cognitive development [61,62]. Social stratification was also evident: children from Scheduled Tribes and Scheduled Castes scored lower than those from higher castes, patterns that mirror caste-based inequities in stunting and service access [43,44,63]. Household wealth, sanitation, and water quality were positively associated with scores, reinforcing the role of socioeconomic and environmental health conditions in shaping learning [40,41,44].

The mediation analysis indicated that the pathways through which ANC is linked to cognition are selective. At age 15, maternal and paternal education partially mediated the association between ANC and Math, while no mediation was observed for PPVT or through mid-day meals. This suggests that numerical reasoning remains more responsive to sustained parental support and structured learning inputs, while verbal ability stabilizes earlier [23,29]. Although mid-day meals did not mediate the ANC and cognition pathway, their programmatic role in supporting attendance and nutritional security remains important in resource-constrained settings [21,64].

These findings have significant implications for policymaking. Although ANC alone cannot fully address cognitive disparities, it serves as a vital early health-system entry point and a proxy for broader engagement with maternal and child health services. However, the attenuation of its effects by adolescence highlights that ANC’s developmental benefits require sustained support through complementary interventions, particularly in nutrition, education, and household environments, to be fully realized.

This is particularly relevant in India, where national ANC coverage improved modestly from 37% in 2006 to 51.2% in 2016. But significant regional disparities persist: in Andhra Pradesh, ANC coverage declined from 76.3% to 67.5% during this period. The COVID-19 pandemic further disrupted service continuity [65–67]. Improving ANC coverage, therefore, requires not only alignment with WHO’s recommendation of eight contacts per pregnancy but also embedding it within a multisectoral strategy that links maternal health services with early education, school nutrition, social protection, and environmental health.

Equity is of crucial importance. Caste-based inequities in maternal health service access, combined with poverty and gender disparities, continue to influence developmental opportunities in India. Universal school enrolment, particularly for girls and disadvantaged groups, and improved environmental health conditions are essential to sustaining cognitive gains. Tackling these inequities is essential to breaking intergenerational cycles of deprivation. In this context, ANC functions not only as a protective factor but also as a gateway to broader developmental pathways, particularly when integrated with targeted nutrition and parental support interventions [13,63,64].

In conclusion, these findings align with the “fetal origins hypothesis” [68], which underscores that early life conditions are associated with later health and development. Although the associations of many covariates became modest as children aged, disadvantaged children tended to accumulate risks over time, reinforcing disparities [69,70]. This cumulative burden highlights the risk of widening gaps when inequities emerge early. Children born into better-resourced households may avoid cycles of poverty and undernutrition, while those from marginalized groups remain vulnerable to intergenerational disadvantage. ANC is thus best interpreted as an early marker of investment in children’s development that must be integrated into broader, equity-driven strategies to achieve sustained and inclusive improvements in cognitive outcomes.

### 5.1 Limitations of this study

The first limitation of this study is the absence of data on the availability of health services and the geographical proximity of healthcare services to those in need. This constraint has impeded a comprehensive examination of contextual determinants influencing healthcare utilization and equity.

Another constraint was the reliance on only two cognitive assessments, PPVT and Math scores, which limited the scope of outcome measurement and may have excluded other dimensions of cognitive development. While we adjusted for school enrolment, maternal education, and household wealth, PPVT and Math scores may be strongly influenced by exposure to formal schooling, which could affect the interpretation of the results. For these reasons, our results are presented as statistical associations rather than causal effects. Thus, the scores should be interpreted as proxies of verbal and numerical reasoning, rather than comprehensive measures of cognitive ability.

Thirdly, the Young Lives sample was intentionally pro-poor and, in the Indian context, restricted to the state of Andhra Pradesh. This purposive sampling strategy, while valuable for understanding disadvantaged populations, limits the generalizability of findings to broader national contexts. Overrepresentation of socioeconomically disadvantaged groups may also constrain the external validity of the results.

The fourth limitation of the study is that, despite adjusting for multiple covariates, residual confounders such as preferential parental investment or unmeasured differences in school quality may have influenced the test outcomes in the Young Lives study.

Fifthly, mediation analyses in this study were restricted to parental education and access to mid-day meals. These factors were selected a priori based on existing literature [23,29] that identifies them as plausible pathways through which associations between ANC and children’s cognitive outcomes may operate. Other covariates included in regression models, such as caste, household wealth, or household environment characteristics, were treated as background determinants or adjustment variables. Although these factors are closely linked to both ANC and child development, they were not theorized as mediators within the scope of this analysis. Instead, they were incorporated as covariates to account for structural and socioeconomic variation, rather than being formally tested in mediation models.

Finally, the ‘high ANC’ category used in this study was based on a composite measure constructed by the Young Lives research team, which included the timing of the first ANC visit, the total number of visits, and receipt of TT injections. While this measure captures service quantity and timing, it does not include quality-related aspects such as counseling, provider competence, or supplementation. The absence of quality indicators is a limitation in the interpretation of our findings related to ANC.

## Supporting information

S1 Table STROBE Statement Checklist of items that should be included in reports of cohort studies, detailing where each criterion is addressed in the manuscript. (PDF)

S2 Table Supplementary table with direct, indirect, and total effects from the mediation analysis at age 15. (PDF)
