# Supplementary material for: The early advantage: How antenatal care shapes cognitive development in India: Evidence from Young Lives, India
Source: PLOS Glob Public Health. 2025 Nov 26;5(11):e0004801. doi: 10.1371/journal.pgph.0004801 (PMC12654890; doi:10.1371/journal.pgph.0004801)
Supplement: S1 Table — (PDF) [file pgph.0004801.s001.pdf]

This checklist is based on the STROBE (Strengthening the Reporting of Observational Studies in Epidemiology) Statement				
**Section**	**Item No.**	**Recommendation**	**Page No.**	**Relevant text from manuscript**
**Title and abstract**	1	(a) Indicate the study's design with a commonly used term in the title or abstract	2	Drawing on data from the Young Lives study, this research examines the developmental trajectories of 1,918 children, who were tracked longitudinally from age 1 to 15.
		(b) Provide in the abstract an informative and balanced summary of what was done and found	2	Abstract provides a concise overview of background, objectives, analytical approach (multilevel regression and PSM), and key findings.
**Introduction**	2	Explain the scientific background and rationale for the investigation being reported	3-5	Introduction, paragraphs 1-4 and paragraph 7 describe the rationale for examining antenatal care (ANC) and children's cognitive outcomes.
	3	State specific objectives, including any prespecified hypotheses	5	This study seeks to fill these gaps by examining the association between maternal ANC and children's cognitive test scores, specifically the Peabody Picture Vocabulary Test (PPVT) and Math scores.
**Methods**	4	Present key elements of study design early in the paper	8	This study utilizes secondary data from the Indian sample of the younger cohort in the Young Lives longitudinal survey, encompassing children, their households, and communities. Data were collected across five survey rounds, conducted in 2002, 2006, 2009, 2013, and 2016, covering developmental trajectories and contextual influences.
	5	Describe the setting, locations, and relevant dates, including periods of recruitment, exposure, follow-up, and data collection	8-9	Data were collected across five survey rounds, conducted in 2002, 2006, 2009, 2013, and 2016… Data collection was undertaken in the states of Andhra Pradesh (Coastal Andhra and Rayalaseema regions) and Telangana. The 'index child'… was aged, on average, 2, 5, 8, 12, and 15 years during rounds 1 through 5
	6	(a) Cohort study—Give eligibility criteria, and sources/methods of selection of participants	9-10	For this paper, we restrict all analysis to the children from the Indian sample of the younger cohorts, as a large set of information on child and maternal characteristics, vaccination status, and whether the mother received ANC is available only for this group. Other relevant covariates, including those related to early childhood cognitive development, which form the crux of our analysis, are available only for the younger cohort. These indicators reflect the

				initial developmental and contextual conditions of individual children and were used to frame the analysis of the Younger Cohort within the longitudinal study. Data were not available on child characteristics or important initial variables influencing the older cohort's child growth and well-being.
	7	Clearly define all outcomes, exposures, predictors, potential confounders, and effect modifiers	10-13	Outcomes: Children's cognitive outcomes were measured using the Peabody Picture Vocabulary Test (PPVT) and Math assessments, based on raw PPVT and standardized Math scores at ages 5, 8, 12, and 15.<br><br>Exposure: The main exposure, maternal antenatal care (ANC) utilization, was measured as both a binary variable (any ANC vs. none) and a four-level ordinal variable (no, low, medium, high utilization).<br><br>Predictors/covariates: Maternal and paternal education (in completed years of schooling), child's sex, caste group, household wealth index, sanitation, and water source.<br><br>Potential confounders: Household wealth, maternal education, and caste were included as potential confounders to reduce bias in observed associations.
	8	For each variable, give sources of data and methods of assessment; describe comparability if multiple groups	10-11	Data were derived from Young Lives survey instruments administered to caregivers of children. PPVT and Math tests were culturally adapted and validated through translation, pilot testing, and expert review. Cognitive scores were standardized as z-scores for comparability across waves.
	9	Describe any efforts to address potential sources of bias	23-25 and 31	Propensity Score Matching (PSM) was employed to minimize selection bias due to observed confounders and multilevel model accounted for clustering at household and community levels to reduce contextual bias.
	10	Explain how the study size was determined	23-25	Analyses used the full younger cohort (n = 2000). The study did not employ an a priori power calculation, as it utilized all available longitudinal data for the Indian sample.

	11	Explain how quantitative variables were handled in the analyses	10-14	All key variables were defined and coded based on standardized Young Lives survey instruments and accompanying derived datasets. The Young Lives research team constructed several analytic variables and applied harmonized coding procedures to ensure comparability across survey rounds. Continuous cognitive scores (PPVT and Math) are given by Young Lives, allowing comparison over time. Maternal antenatal care (ANC) was coded both as a binary indicator (any ANC = 1, none = 0) and as a four-level ordinal variable (no, low, medium, high utilization) to capture graded associations. The household wealth index, computed from housing quality, durable goods, and facilities, was converted into quintiles to represent relative socioeconomic position. These standardized and coded variables were used directly in the analyses, ensuring internal consistency and replicability across the longitudinal dataset.
	12	(a) Describe all statistical methods, including confounder adjustment	14-15	Analyses were conducted using multilevel regression models and PSM to examine associations between maternal ANC and children's cognitive outcomes. Multilevel models accounted for the nested data structure (child, household, community) and adjusted for key sociodemographic confounders, including maternal and paternal education, child's sex, caste, place of residence, household wealth, sanitation, and water source. PSM was applied as a robustness check to minimise selection bias from observable covariates, using nearest-neighbour matching with three neighbours. Additionally, **mediation analysis** was conducted to explore plausible pathways through which ANC may be associated with children's cognitive outcomes, focusing on two mediators: parental education and access to mid-day meals. These factors were selected a priori based on prior literature and tested using regression-based mediation models that decomposed the total association into direct and indirect effects. All analyses were performed using STATA Version 17.
		(b) Describe subgroup/interaction analyses	-	No separate subgroup or interaction analyses were undertaken. However, gender and place of residence were included as covariates in all regression models to account for their potential influence on

				the associations between maternal ANC and children's cognitive outcomes.
		(c) Explain how missing data were addressed	15	Observations with incomplete information on key exposure (ANC) or outcome variables (PPVT and Math scores) were excluded from the analysis. The final analytic sample, therefore, includes only participants with complete data on these core variables.
		(d) Cohort study—Explain how loss to follow-up was addressed	9	One of the strengths of the YL study is that even by the fifth round, the overall attrition rate was only about 5 percent over nearly 15 years, which can be regarded as one of the lowest in longitudinal surveys of this nature
		(e) Describe any sensitivity analyses	14	Sensitivity analyses were not conducted for this study. The analysis focused on multilevel regression; Propensity Score Matching models and mediation analysis as primary and robustness approaches to examine associations between ANC and cognitive outcomes.
**Results**	13	(a) Report numbers of individuals at each stage (eligible, included, analysed)	15	The final analytic sample included 1,985 children from the younger cohort with complete data on maternal ANC exposure and cognitive outcomes across the survey rounds.
	14	(a) Give characteristics of participants (demographic, social, etc.)	15	**Table 1** presents the descriptive characteristics of the study population, including child sex, maternal and paternal education, caste, household wealth, sanitation, water source, and residence (rural/urban). These variables summarize the socioeconomic and demographic profile of the sample used in analyses.
	15	Report outcome data over time	23-25	**Table 2** presents mean Peabody Picture Vocabulary Test (PPVT) and Math scores across ages 5, 8, 12, and 15, stratified by maternal ANC status (none vs. any ANC).
	16	(a) Give unadjusted and adjusted estimates, with 95% CI, and specify confounders	23-25 and 33	**Table 2** presents adjusted estimates from multilevel regression models examining the association between maternal ANC access and children's cognitive outcomes (PPVT and Math scores) at ages 5, 8, 12 and 15. All models adjust for child's sex, caste, household wealth, sanitation, drinking-water source, maternal BMI, preterm birth, maternal and paternal education, school enrolment, and place of residence. Statistical significance levels are shown with standard errors, and 95% CIs are reported for mediation results in the manuscript. Only adjusted estimates are shown because the analysis

				focuses on multivariable associations controlling for confounders.
	17	Report other analyses (e.g., mediation, subgroup)		Mediation analyses at age 15 examined the indirect effects of parental education and mid-day meal access on Math and PPVT scores, identifying significant indirect effects through maternal and paternal education but not through mid-day meals. Sensitivity analysis was not performed; however, observations with incomplete ANC or cognitive-score data were excluded after confirming no systematic differences between excluded and retained cases. Descriptive checks indicated no evidence of selective attrition bias.
**Discussion**	18	Summarize key results with reference to objectives	33-35	This study found that maternal access to antenatal care was positively associated with children's cognitive outcomes, particularly in middle childhood. The most consistent differences appeared at age 8, when children whose mothers accessed ANC had higher PPVT and Mathematics scores. The attenuation observed in adolescence suggests that the influence of ANC is most visible during early schooling.<br>Although ANC alone cannot close cognitive gaps, it represents a critical early health-system entry point whose developmental benefits must be reinforced through sustained investments in nutrition, education, and household environments.
	19	Discuss limitations and potential bias	36-37	This study is subject to several limitations, including the absence of data on service proximity, reliance on two cognitive tests (PPVT and Math), and the possibility of unmeasured confounding factors, such as parental investment or differences in school quality. The Young Lives sample was pro-poor and restricted to Andhra Pradesh, which may limit generalisability. Results are presented as statistical associations rather than causal effects.
	20	Provide an overall cautious interpretation, considering limitations and similar studies	35	Although ANC alone cannot fully address cognitive disparities, it serves as a vital early health-system entry point and a proxy for broader engagement with maternal and child health services. However, the attenuation of its effects by adolescence highlights that ANC's developmental benefits require sustained support through complementary interventions, particularly in nutrition, education, and household environments, to be fully realized.

	21	Discuss generalisability (external validity)		The Young Lives sample was intentionally pro-poor and, in the Indian context, restricted to the state of Andhra Pradesh. This purposive sampling strategy, while valuable for understanding disadvantaged populations, limits the generalizability of findings to broader national contexts. Overrepresentation of socioeconomically disadvantaged groups may also constrain the external validity of the results.
**Other information**	22	Give source of funding and role of funders		This analysis uses publicly available secondary data from the Young Lives study, funded by FCDO (UK).

Licensed under Creative Commons Attribution 4.0 International (CC BY 4.0)

Please cite as:
von Elm E, Altman DG, Egger M, Pocock SJ, Gøtzsche PC, Vandenbroucke JP; STROBE Initiative.
The Strengthening the Reporting of Observational Studies in Epidemiology (STROBE) statement: guidelines for reporting observational studies.
PLoS Med. 2007 Oct 16;4(10):e296. PMID: 17941714.
