# Supplementary material for: The early advantage: How antenatal care shapes cognitive development in India: Evidence from Young Lives, India
Source: PLOS Glob Public Health. 2025 Nov 26;5(11):e0004801. doi: 10.1371/journal.pgph.0004801 (PMC12654890; doi:10.1371/journal.pgph.0004801)
Supplement: S2 Table — (PDF) [file pgph.0004801.s002.pdf]

Outcome	Mediator variable	NIE (β, 95% CI)	NDE (β, 95% CI)
**Math z-score (Age 15)**	Caregiver Education	0.103 (**0.002, 0.205**)	1.144 (**0.315, 1.971**)
	Partner Education	0.120 (**0.012, 0.228**)	1.246 (**0.399, 2.093**)
	Mid-Day Meal	0.008 (−0.093, 0.109)	1.349 (**0.509, 2.189**)
**PPVT raw score (Age 15)**	Caregiver Education	0.005 (−0.078, 0.088)	−0.381 (−1.448, 0.686)
	Partner Education	−0.028 (−0.193, 0.137)	−0.625 (−2.501, 1.252)
	Mid-Day Meal	−0.001 (−2.284, 2.283)	−0.617 (−2.497, 1.264)

NDE = Natural Direct Effect; TE = Total Effect.
Significance is based on 95% confidence intervals ($p < 0.05$). All models include treatment–mediator interaction and covariate adjustment.

TE (β, 95% CI)
1.246 (**0.397, 2.096**)*
1.366 (**0.515, 2.217**)*
1.357 (**0.518, 2.196**)*
−0.376 (−1.440, 0.688)
−0.652 (−2.532, 1.227)
−0.617 (−3.476, 2.242)
